# Spontaneous emergence of leadership patterns drives synchronization in complex human networks

**DOI:** 10.1038/s41598-021-97656-y

**Published:** 2021-09-15

**Authors:** Carmela Calabrese, Maria Lombardi, Erik Bollt, Pietro De Lellis, Benoît G. Bardy, Mario di Bernardo

**Affiliations:** 1 Department of Electrical Engineering and Information Technology, University of Naples Federico II, Naples, 80125 Italy; 2 EuroMov Digital Health in Motion, University of Montpellier IMT Mines Ales, Montpellier, 34090 France; 3 Center for Robotics and Intelligent Systems (CRIS), Italian Institute of Technology (IIT), Genoa, 16163 Italy; 4 Electrical and Computer Engineering ECE Department and the Clarkson Center for Complex Systems, Clarkson University, Potsdam, NY 13699-5815 USA

**Keywords:** Human behaviour, Applied mathematics

## Abstract

Synchronization of human networks is fundamental in many aspects of human endeavour. Recently, much research effort has been spent on analyzing how motor coordination emerges in human groups (from rocking chairs to violin players) and how it is affected by coupling structure and strength. Here we uncover the spontaneous emergence of leadership (based on physical signaling during group interaction) as a crucial factor steering the occurrence of synchronization in complex human networks where individuals perform a joint motor task. In two experiments engaging participants in an arm movement synchronization task, in the physical world as well as in the digital world, we found that specific patterns of leadership emerged and increased synchronization performance. Precisely, three patterns were found, involving a subtle interaction between phase of the motion and amount of influence. Such patterns were independent of the presence or absence of physical interaction, and persisted across manipulated spatial configurations. Our results shed light on the mechanisms that drive coordination and leadership in human groups, and are consequential for the design of interactions with artificial agents, avatars or robots, where social roles can be determinant for a successful interaction.

## Introduction

Synchronization is a common feature in many human activities from sports to dancing and music playing^1–3^. It is also a typical collective behaviour in many animal groups, from schools of fish and flocks of birds to insect swarms^4–9^.

Synchronization in complex human networks has been the subject of recent work that showed the influence on its emergence of the coupling structure between individuals in a group^10,11^, even after perceptual contact is interrupted^12^, as well as of the coupling strength and presence of delays in the interaction. For example, certain coupling networks such as the all-to-all graph or the star graph were found to be beneficial to achieve synchronization^10,12^ as is the ability of each player to ignore a coupled player and adjust its motion features^11^. Movement coordination was also shown to be instrumental in driving better social interaction and in signaling social affiliation between members of a group performing a joint task^13–15^.

To date no systematic study was carried out to understand, in a controlled set-up, how influential each player is in steering the dynamics of a complex human network towards synchronization despite leadership emergence having been highlighted as a crucial phenomenon to understand such behaviour in the animal world^9,16–20^.

In psychology and organization management, theoretical and empirical studies have focused on leadership emergence mostly from an abstract and language-based signaling perspective. Influence signaling is assumed to flow during social interactions by means of information exchanged through abstract language, whereby personal attributes may promote the election of a leader in the group^21^, and the diversity among individuals in cultural and social attitudes toward cooperation and fairness are crucial for the emergence of communities^22^. In this article, we refer to a more primal, non-verbal and embodied form of interaction, in which leadership emerges out of motor coordination among the individuals in the group. The goal is to further improve the multi-disciplinary understanding of leadership as an inner natural trait characterizing all animal groups where communication does not necessarily rely on language^23–26^.

In the last decades, the necessity became apparent of carrying out a quantitative analysis of leadership^20^, thereby enabling the validation of alternative theories for its emergence^27^. In this context, computer modeling has become an essential tool in many areas of social science^28,29^. For instance, Hubler and Pines^30^ employed analytical calculations and computer simulations to quantify adaptation in a complex system, linking it to the emergence of collective behaviours and leadership. Here, we use data from a controlled experimental set up to investigate quantitatively the emergence of leader-follower relationships in a group of people performing a coordinated oscillatory task, an abstraction of the motion of rocking chairs or violin players used as paradigmatic examples in^2,11^. By combining phase computation with information theoretic measures such as causation entropy, we infer causality by extracting the directional information flows from the position (or phase) time-series acquired from the group members^31,32^. We found that leadership spontaneously emerges as an organizing phenomenon steering and enhancing group coordination. More strikingly, we unfolded three fundamental patterns through which leadership can appear in a group; a pattern where the leader, i.e., the most influential group member, is the person moving ahead of all the others (phase lead), pattern where the leader is the person moving behind all of them (phase lag), and one where leadership is shared between the two individuals bracketing group motion, i.e., leading ahead and lagging behind the other participants.

We repeated the experiment via Chronos^33^, a recently developed digital platform allowing players to participate remotely to the experiment via a computer terminal equipped with a leap motion controller^34^. Again, via a completely different set-up, we found that leadership emerges and enhances group coordination. Also, this happens via the same leadership patterns confirming that they persist even when communication between the group members is digitally mediated.

Our results reveal that leadership emergence is a crucial phenomenon that organizes the dynamics of complex human network performing a joint oscillatory task, such as violin players studied in earlier work^11^. Moreover, such an important emerging property is found to occur persistently in three major different patterns where the most influential player(s) is/are not always the one/s leading in phase. Our findings shed light on the mechanisms that drive coordination in human groups and can be influential not only for a better understanding of human embodied interactions, but also for the design of interactions with artificial agents, avatars or robots, where social roles can be instrumental to enhance coordination.

**Figure 1:**
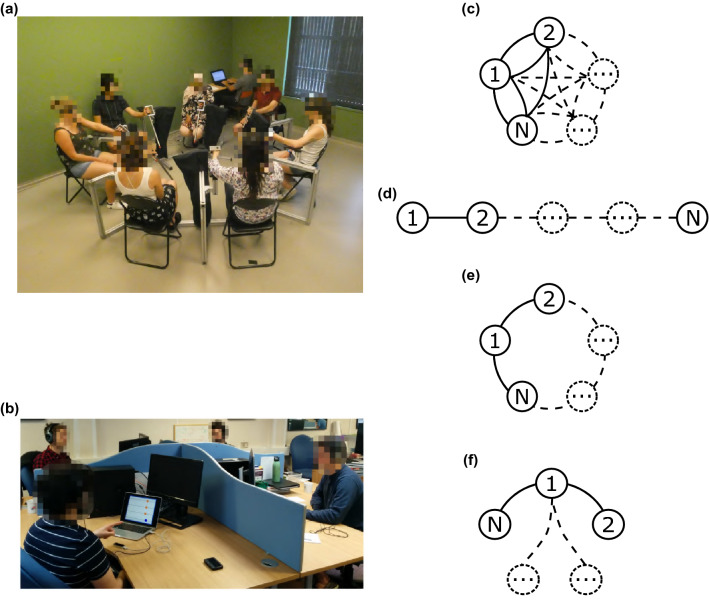
Experimental setup. (**a**) The panel describes the first setup (*Experiment 1: pendula*), where each player was asked to move a pendulum with their own preferred hand and try to synchronize its movement with the others (see “Methods” for more details). (**b**) The second setup (*Experiment 2: Chronos*), where participants had to oscillate and synchronize the index finger of their preferred hand over a Leap Motion controller (Leap Motion, Inc.^34^) while being virtually connected with the others through the platform Chronos^33^. The main difference with the previous setup is the absence of visual and acoustic coupling among the players due to physical separators and headphones, so as to cancel or minimize social interactions. (**c**–**f**) We plotted the four configurations (*topologies*) implemented in the experiments: *complete graph* (**c**), *path graph* (**d**), *ring graph* (**e**), and *star graph* (**f**), with *N* being the number of players involved in the experiments. In the *complete graph*, each participant can see all the others; in the *path graph*, every player can see the trajectories of two neighbours with the exception of the two external participants (the head and tail of the chain) that are constrained to visualize the motion of only one neighbour; in the *ring graph*, each player can see the motion of only two neighbours; in the *star graph*, all the players observe the motion of one central player who, conversely, sees the motion of all the others.

**Figure 2:**
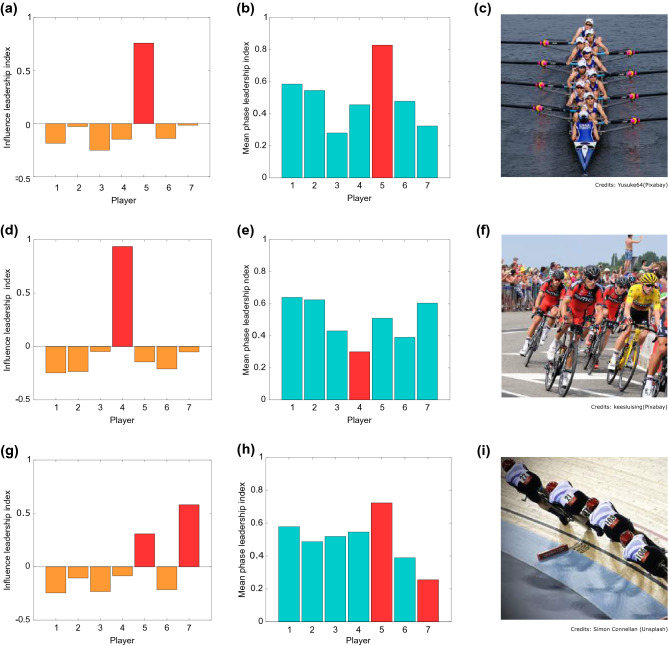
Description of the leadership scenarios. In the first column, each bar represents the value of the influence leadership index (see “Methods”). The central column shows the mean phase leadership index for each player. The red bars identify the relationship between these two metrics typical of each pattern (Pattern 1- top row, Pattern 2- middle row, Pattern 3- bottom row). The third column displays an example for each leadership scenario from sports world. In particular, (**c**) reports an example of the first scenario, where the role of a coxswain is to steer the boat and coordinate the rhythm of the team members. (**f**) Depicts professional road cyclists racing at the *Tour de France*. It is a paradigmatic example of leadership assigned to the participant lagging behind all the others, since the team leader typically drafts the wheels of her/his teammates (domestiques) but is pivotal for determining the team strategy. (**i**) An example of shared leadership observed during team pursuit in track cycling, where the fastest player in front sets the pace, but also accounts for the position of the third lagging player, since the final time for the team is taken when the third team member crosses the finish line. Images are taken from https://pixabay.com and https://unsplash.com.

## Results

*Leadership patterns* We carried out a set of dedicated experiments where a group of participants was asked to oscillate a manipulandum (or their index finger) while synchronizing their motion with each other (see Fig. 1 and “Methods” for further details).

Data was analysed to extract the phases (or positions) of the players’ motion and compute two metrics to assess their leader/follower roles. Namely, an influence index based on the computation of causation entropy, and a phase leadership index based on the phase analysis of the time series of the players’ motion (see “Methods” for further details).

**Figure 3:**
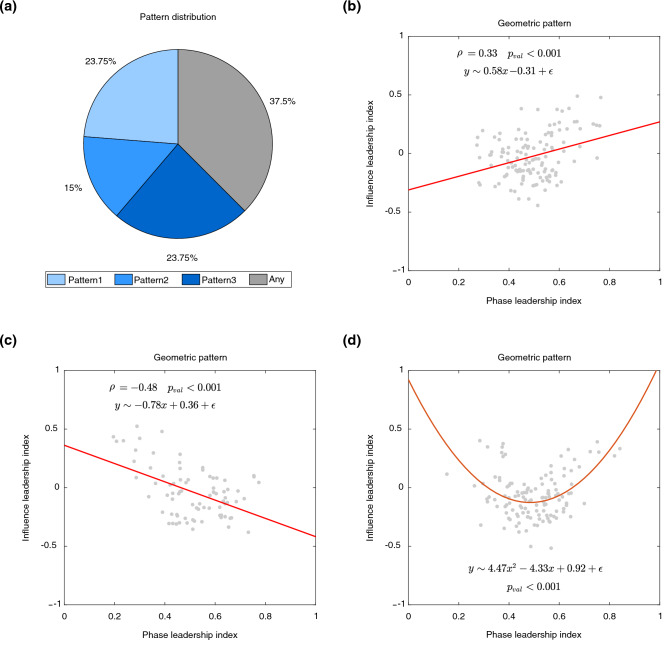
Distribution and characterization of the leadership scenarios. (**a**) The pie chart depicts how the three patterns were distributed among the trials of Experiment 1. A correlation analysis between the net information flow NetCaus and the mean position ranking ${{\overline{H}}}$ in the trials where Pattern 1 and 2 were observed is reported in (**b**) and (**c**), respectively. The linear fitting is represented by a red solid line. In Pattern 3, the relationship between NetCaus and ${\overline{H}}$ is instead captured by a parabolic curve fitting, which is the red solid line in (**d**).

**Figure 4:**
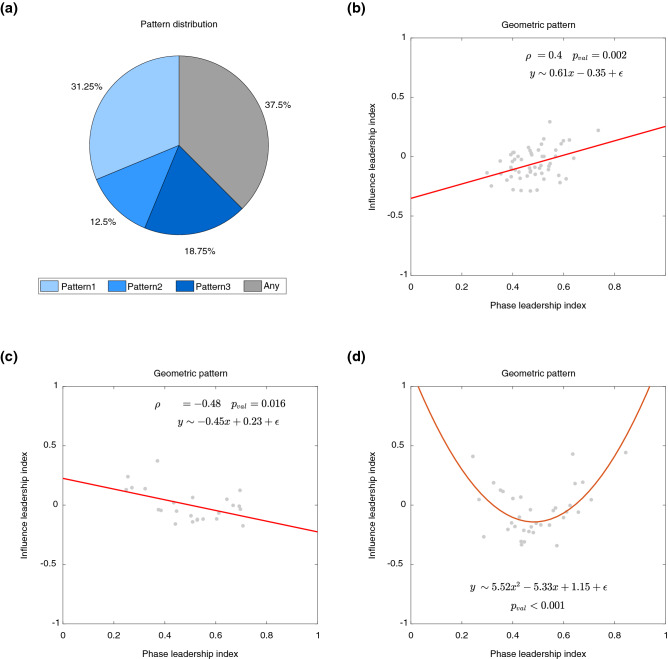
Distribution and characterization of the leadership scenarios in absence of social interaction. (**a**) The pie chart depicts how the three scenarios were distributed among the trials of Experiment 2. A correlation analysis between the net information flow NetCaus and the mean phase ranking ${{\overline{H}}}$ in the trials where Pattern 1 and 2 were observed is reported in (**b**) and (**c**), respectively. The linear fitting is represented by a red solid line. In Pattern 3, the relationship between NetCaus and ${\overline{H}}$ is instead captured by a parabolic curve fitting, which is the red solid line in (**d**).

As shown in Figs. 2 and 3, we found that in over 60% of all trials, one out of three different leadership patterns clearly emerged.

In Pattern 1—“the fastest leads”—the individual who was the most influential, as assessed by computing the causation entropy between each pair of group members, was also the one leading the group motion in phase (see Fig. 2a, b). In Pattern 2—“the slowest leads”—the most influential player was the one lagging behind all the others in phase, see Fig. 2d–e. Finally, Pattern 3—“shared leadership”—was one where the highest influence was shared by two players with the highest and lowest phase leadership index respectively.

The three leadership patterns we uncovered have analogues in configurations often found in nature and sports. For example, Pattern 1 is the one found in rowing competitions where rowers are driven and motivated by the coxswain, see Fig. 2c. Pattern 2 resembles that used by teams in road cycling where partners work for the benefit of the team leader, who, although moving behind all the domestiques, determines the pace of the group, see Fig. 2f. Pattern 3 is the same pattern emerging in track cycling where during team pursuit rounds the success of the group is influenced not only by the team member in the front position who sets the pace (*phase leader*) but also by the cyclist in the third position, who determines the final time by crossing the finish line, see Fig. 2i.

A geometric interpretation of these three patterns can be obtained by plotting the net predicted information flow against the mean ranking in phase for each of the three leadership scenarios, as shown in Fig. 3.

A linear fitting shows that, in the trials where the phase leader emerges as the most influential (Pattern 1), ranking in phase and influence are positively correlated [$r(19)=0.33$, $R^{2}=0.11$, $p<0.001$]. Instead, when leadership is assumed by the player most lagging in phase (Pattern 2), the opposite is observed [$r(12)=-0.48$, $R^{2}=0.23$, $p<0.001$]. Interestingly, a linear fitting [$R^{2}=0.02$, $p=0.17$] cannot capture Pattern 3, where two players share leadership, and a parabolic interpolation is required to depict the coexistence of lagging and phase leaders [$R^{2}=0.24$, $p<0.001$].

*Leadership emergence aids coordination* We found that one of the three leadership patterns described above emerged in a significant number of trials but not in all of them. Specifically, we detected one of the three scenarios emerging in $62.50\%$ of all the trials, whereas in the remaining trials no consistent pattern emerged. We ascertained that the frequency of occurrence of one of the three patterns was different from chance by comparing it with the expected frequency of occurrence ($28.57\%$) using a $\chi ^{2}$-test [$\chi ^{2}(3)= 83.54$, $p<0.001$]. Most importantly, we found that the level of coordination in the group was significantly higher in those trials where leadership emerged than in those where it did not. In particular, we computed the group order parameter *z* (see “Methods” for further details) to be equal to 0.73 on average when leadership emerged [$M_1=0.73$, $SD_1=0.17$] while being equal to 0.60 otherwise [$M_2=0.60$, $SD_2=0.17$]; such difference being statistically significant [independent *t*-test, $t(78)=3.37$, $p<0.001$, *Cohen’s* $d=0.78$].

*Group structure influences leadership emergence* We manipulated the group interaction structure to assess whether different topologies had an effect on leadership emergence. A $\chi ^{2}$ test revealed that the distribution of the three leadership patterns across the topologies is different from uniform [$\chi ^{2}(3) =10.88$, $p=0.012$]. In particular, we observed a higher occurrence of leadership emergence (via one of the three patterns described in this paper) when all players were interacting with everyone else (complete graph topology, Fig. 1c) or when a central player was interacting with the rest of the group (star graph structure, Fig. 1f, see Tables S2–S3 in Supplementary Information). These coupling configurations are also those that were found in previous work^10,12^ to facilitate the onset of motor coordination in the group [$F(3,76)=31.55$, $p<0.001$, $\eta ^{2}=0.55$].

*Leadership patterns persist in the absence of physical interaction* We repeated the experiment in the digital world without physical interaction, by means of Chronos, a software platform allowing remote coordination between players^33^, see Fig. 1b and “Methods” for more details on the experimental set-up. We found again that, even in the absence of direct visual/auditory coupling among the players, one of the three leadership patterns emerged in $62.50\%$ of the trials, see Fig. 4a; a $\chi ^{2}$-test confirming that the frequency of occurrence of the leadership scenarios was different from chance [$\chi ^{2}(3)= 18.81$, $p<0.001$].

Again, the average order parameter in the trials where these patterns are observed was higher than that observed in the remaining trials [$M_1=0.68$, $SD_1=0.16$ versus $M_2=0.55$, $SD_2=0.14$], and this difference was found to be significant [independent *t*-test, $t(30)=2.23$, $p=0.03$, *Cohen’s* $d=0.82$]. Also, the geometric interpretation of the three scenarios in this second experiment was qualitatively the same as in the experiments where players were physically present in the same room, see Fig. 4b–d.

## Discussion

We analyzed data from controlled experimental set-ups where a group of individuals was asked to perform an oscillatory task synchronizing its motion with that of others in the group.

We found that leadership emerges spontaneously in complex human networks as an organizing mechanism facilitating synchronization, even when coupling among the players is digitally mediated. Three different patterns were detected through which leadership emerged. One, where the most influential player is the one ahead in phase, another, where the lagging one is the most influential, and the third, where a shared leadership arrangement emerges between the players furthest ahead or lagging in phase.

Our results show that, as noticed for groups in the animal world, the emergence of leadership roles is a natural phenomenon occurring in human groups even when it is not solicited from external instructions or assignments.

The *shared leadership* pattern that we observe in about 23% of all cases has been highlighted as relevant in neuroscience and psychology. For instance, the principles of psychomotor games in groups of children, illustrated in^35^, emphasize the role played by two main *actors* during the interaction, which are defined therein as the *leader*, and the *complementary leader*. These roles are not pre-assigned but are observed to emerge during the interaction and to coexist, contributing to the fulfillment of new functional needs when required. The cooperation of different leaders has also been remarked as being beneficial in public security. Mixing different leader types, placing some in the immediate proximity of the crowd and others scattered around the environment, ensures better safety of the public, providing the best effectiveness on crowd confinement^36^. A similar type of shared leadership was also detected in walking groups where the emergence of two coexisting (and diversely specialized) leaders was suggested^37^.

Additionally, previous research revealed the significant impact of shared leadership on team creativity and effectiveness^38,39^. It was noted that the existence of different but cooperating leaders enhances teams’ ability to cooperate and perform reliably in urgent, unpredictable, interdependent contexts while also being beneficial to build novice team members’ skills^40^. This has also been detected in the animal kingdom where having two leaders instead of one has been found to be a necessary and sufficient condition to guide groups of animals to avoid a predator, as for instance in direct observations of a school of fish^41^.

Our study can be extended in several directions. Indeed, preliminary observations suggest that these leadership patterns emerge more frequently in certain interaction structures (the complete and star graph topologies)—see the Supplementary Information for more details. Furthermore, the complete and star graph arrangements of the group are also characterized by the prevalence of the shared leadership pattern above the others. A reasonable explanation of the link between leadership emergence and the star and complete graphs is that these two topologies are known to favour synchronization in human groups^11,12^, but further evidence is required to confirm this hypothesis.

Furthermore, our experimental results can also unveil important information about successful strategies adopted for the prevalence of specific agents and the emergence of coordination during social interactions. Future investigation will focus on the understanding of the evolution of the coordination behavior while the interaction proceeds and of the influence of the playing sequence over the different topologies on the synchronization results.

Our contribution perfectly matches the interest towards the expanding computational research in complex social network theory^42^ and human interaction dynamics^43^ that leverages the use of quantitative and computational methods to model, analyze, and interpret the mechanisms by which cooperation among humans evolves^44^. Spontaneous collective behaviour emerges from individual internal decision making processes, each agent acting according to her/his own sensitivity to social opinion and group dynamics^42,45^. Our unfolding of different leadership patterns represents a novel tool for to analyse individual strategies adopted in a group and to understand what motivates some players to behave as selfish agents or as resilient cooperators^43^.

Following this, the insight we gain on how leaders influence the motion of a group might be valuable in human-robot interaction, and specifically when mixed groups of humans and robots need to coordinate to perform a joint task^46^. Indeed, the leadership patterns that we highlighted might be used to program the motion of one or more artificial agents to enhance the performance of the human-robot group. This could be relevant, for instance, to aid the design of innovative rehabilitation procedures in those impairments where the imitation and movement synchronization mechanism fail^47–50^.

## Methods

### Participants

- *Experiment 1: pendula* Twenty-eight right-handed students at the University of Montpellier took part in the experiment. Participants were asked to move their pendula in synchrony with the others, see Fig. 1a.
  Participants were distributed in four groups of seven individuals, each as follows:
  - group 1 (G1)- mean age = 25.6 y ± 3.1, 5 females, 2 males;
  - group 2 (G2)- mean age = 22.4 y ± 2.4, 6 females, 1 male;
  - group 3 (G3)- mean age = 20 y ± 2.6, 3 females, 4 males;
  - group 4 (G4)- mean age = 21.9 y ± 2.27, 4 females, 3 males.
  These data were collected in the study of Bardy et al.^12^, and have been re-analysed here in terms of leadership emergence. Please, see the reference for further details.
- *Experiment 2: Chronos* Two groups of six participants, each recruited from an initial pool of forty volunteers, employees and students at the EuroMov center and at University of Montpellier, took part in the experiment (all right-handed). They had no expertise in typical sensori-motor synchronization activities (music or dance). Volunteers were divided in two groups as follows:
  - group 1 (CG1)- mean age = 22.5 ± 4.04, 3 females, 3 males;
  - group 2 (CG2)- mean age = 26 ± 12.07, 4 females, 2 males.
  Both studies were carried out according to the principles expressed in the Declaration of Helsinki, and was approved by the local ethical committee (EuroMov IRB $\#1811A$ and IRB $\#1901B$, University of Montpellier). All participants provided their written informed consent to participate in the study, and such consent was also approved by the ethical committee. In addition, all volunteers also provided informed consent to publish identifying images.

### Experimental setups

The groups of participants listed above were involved in two different types of experiments as described in what follows.

- *Experiment 1: pendula* Participants were distributed in 4 groups of 7 individuals each. Participants were asked to sit in a circle in a quiet room with no distractions, and each of them was instructed to move a pendulum back and forth in a synchronized way with the others. A demonstration was performed to make sure the task was understood by each participant. Each group performed the experiments in four different configurations, corresponding to the interaction patterns depicted in Fig. 1c–f. These interaction topologies were implemented by asking participants to wear a pair of goggles made ad hoc, see Figure S1 in the Supplementary Information, that allowed to restrict the field of vision of each participant and hence define the other participants with whom they were in visual contact during the trial. For every different configuration, each of the 4 groups performed 5 trials of 90 s each.
- *Experiment 2: Chronos* Participants were connected through the hardware/software platform Chronos developed in previous work by some of the authors. As described in^33^, the set-up consists of input/output devices, a centralized unit processing data, broadcasting movement information to the players according to the desired network structure being implemented, and a Wi-Fi apparatus connecting all the components together. The movements of each participant are detected by a low-cost position sensor^51^, and individuals interact with each other through their own personal computer, on whose screens the central unit broadcasts the appropriate position trajectories according to the assigned topology (see Figure S2 in Supplementary Information). Specifically, participants were distributed in 2 groups of 6 individuals each, and were asked to move their index finger on a leap motion controller so as to move a ball on the screen representing their own avatar, oscillating from left to right and vice versa, in a synchronized way with the others. Namely, they were instructed to *Synchronize the movement of your finger from left to right with the movement of the others, as naturally as possible, as if you could do it for 30 minutes*. A demonstration was performed to make sure the task was understood by each participant. The Chronos software allowed to manipulate the structure of the information shared on the screen of each player so as to implement the 4 different interaction topologies depicted in Fig. 1c–f. Moreover, participants were separated by barriers and wore headphones playing white noise. In each configuration, volunteers performed 4 trials of 75 s each.

In both the experiments, each trial was divided into three different parts:

- *Eyes-closed condition 1* Volunteers were first asked to swing the pendulum with their preferred hand at their own comfortable tempo during 30 s (Experiment 1), while keeping their eyes closed, or to move their own ball in a natural way for 15 s (Experiment 2), without seeing the avatars of the other participants.
- *Eyes-open condition* Players opened their eyes and were asked to synchronize their manipulandums, and possibly to reach and maintain a phase synchronization regime during 30 s (in both Experiment 1 and Experiment 2);
- *Eyes-closed condition 2* Participants closed their eyes again, and were asked to keep on going with the movement for 30 s (both for Experiment 1 and Experiment 2).

In *Experiment 1: pendula* an acoustic signal notified the change between visual conditions whereas in *Experiment 2: Chronos* the balls manipulated by the neighboring players were made to appear/disappear on the players’ screens as needed. For the purpose of this study, which focused on the emergence of leadership in groups of interacting agents, we only considered data from the *Eyes-open condition*.

*Data preprocessing* Before performing data analysis, we preprocessed data.

*Experiment 1: pendula* Potentiometers were used to compute the projection of the hand motions on the floor through a voltage-divider formula. The position time-series were smoothed out through a moving average filter with time window equal to 10 samples, that is, with a time-step $\Delta t= 0.05\,{\text{s}}$.

*Experiment 2: Chronos* The Chronos architecture is a computer-based platform consisting of different hardware/software devices. A central server unit receives position data from the client-players and broadcasts movement information to a subset of the others, according to the desired implemented topology, through a Wi-Fi network. The positions of each agent were captured by a Leap Motion device- a low-cost position sensor- and appear on the screens of each individual personal computer. The position time-series of each player is sampled at 10 Hz. Data were interpolated through a spline to obtain a 100 Hz sampling. Then, the position time-series were filtered through a Butterworth filter with a cutoff frequency equal to twice the typical human natural movement frequency ($\sim 3$ Hz).

For both datasets, the Hilbert transform method^52^ was employed to reconstruct the phase associated to each agent from its position time series.

### Data analysis

For every individual, say *i*, we reconstructed from data the time-series of the phase of their motion. We then determined the phase leadership index, say $H_i(t)$, of that individual in the group at every time instant. Namely, the value of $H_i(t)$ ranges from 1 to 7, with 1 (7) corresponding to player *i* indicates what group members are being followed (led) in phase by all the other player. We then computed the average phase index of individual *i* over the entire trial (${\overline{H}}_i$) to quantify their tendency to lead in phase.

To complement the analysis and uncover who is influencing whom in terms of the information flow between group members, we evaluated next, from the time series of the phases of their motion, the causation entropy between every pair of agents in the group^31^. Then, we computed the net predicted information flow, ${\text{NetCaus}}_i$, from agent *i* to the rest of the group. This index defines the ability of each agent of influencing the motion of the whole group. The combined analysis of the mean phase ranking and of the net predicted information flow across all the groups evidences the three leadership emergence patterns described in this paper.

We describe below how each of the indexes we adopt in our study was defined:

- *Mean phase leadership index* Knowledge of the phase of all the players at each time instant $t_k$ allows to compute the phase difference among every pair of agents, that is,
  1 $$\begin{aligned} \phi _{ij}(t_k)= \theta _i(t_k) -\theta _j(t_k) \quad \forall {\ i,j} =1,\dots ,\ N_{\mathrm{players}}, \quad \forall {\ k} =1,\dots ,\ N_{\mathrm{instants}}. \end{aligned}$$
  where $N_{\mathrm{instants}}$ is the number of time steps of duration $\Delta t$, that is the sampling period, of the trial of duration *T*. We say that player *j* is *ahead* of *i* at time $t_k$ when $\phi _{ij}(t_k)<0$. Then, we denote ${\mathscr {L}}_i(t_k)$ the set of players that are ahead of player *i* at time $t_k$, that is,
  2 $$\begin{aligned} {\mathscr {L}}_i(t_k)= \{ j {\text{|}}\phi _{ij}(t_k) \le 0 \} \quad \forall {\ i} =1,\dots , \ N_{\mathrm{players}}, \quad \forall {\ k} =1,\dots ,\ N_{\mathrm{instants}}. \end{aligned}$$
  The phase leadership index of player *i* at time instant $t_k$, $H_i(t_k)$, is given by the cardinality of the set ${\mathscr {L}}_i(t_k)$, i.e.
  3 $$\begin{aligned} H_i(t_k)=|{\mathscr {L}}_i(t_k)|+1 \quad \forall {\ i} =1,\dots ,\ N_{\mathrm{players}}, \quad \forall {\ k} =1,\dots ,\ N_{\mathrm{instants}}. \end{aligned}$$
  In every trial of each experiment, we compute the mean phase leadership index ${\overline{H}}_{i}$ of player *i* as the time average of $H_i(t_k)$:
  4 $$\begin{aligned} {\overline{H}}_{i}= {\frac{1}{ N_{\mathrm{instants}}}} \sum _{k=1}^{N_{\mathrm{instants}}} H_i(t_k), \quad \forall {\ i} =1,\dots ,\ N_{\mathrm{players}} \end{aligned}$$
- *Net causation entropy* Given a discrete random variable *V*, the Shannon information content associated to *V* can be computed as
  5 $$\begin{aligned} H(V)=- \sum _v p(v)\log {p(v)}. \end{aligned}$$
  where *p*(*v*) is the probability mass function of *V*. *H*(*V*) is called the *Shannon entropy* (from now on, simply *entropy*) associated with *V*^53^.
  Given two (discrete) stochastic processes $X(t_k)$ and $Y(t_k)$, it is possible to define the *transfer entropy*^54^ as:
  6 $$\begin{aligned} T_{X\rightarrow Y}=H(Y(t_k)|Y(t_k-\tau ))-H(Y(t_k)|X(t_k-\tau ),Y(t_k-\tau )). \end{aligned}$$
  $T_{X\rightarrow Y}\ge 0$ represents the information that flows from *X* to *Y* over the time interval $[t_k-\tau ,t_k]$. In other words, transfer entropy measures the uncertainty reduction in inferring the future state of the process *Y* when, in addition to its current state $Y(t_k)$, also the current state $X(t_k)$ of the process *X* is known.
  Transfer entropy successfully detects the directionality of the information flow and, therefore, it may be used to detect causality between two processes. However, when other processes affect both *Y* and *X*, the causal relationship between *X* and *Y* might be poorly related with the transfer entropy^55^. In other words, transfer entropy is a bi-factor measure, so it is unable to distinguish direct versus indirect influences when three or more stochastic processes may be involved. For this reason, in multi-agent systems with more than 2 interacting entities, *causation entropy* is used to neutralize the effect on transfer entropy of the other entities, whose state is encoded in a third (vector) process *Z*(*k*):
  7 $$\begin{aligned} C_{X\rightarrow Y|(Y,Z)}=H(Y(t_k)|Y(t_k-\tau ),Z(t_k-\tau ))- H(Y(t_k)|X(t_k-\tau ),Y(t_k-\tau ),Z(t_k-\tau )). \end{aligned}$$
  This is read as the causation entropy influence as the remaining information flow from $X\rightarrow Y$ conditioned on we already have accepted the value for information flow between *Y* and *Z*. For a discussion about the selection of the delay $\tau$^56^, see the Supplementary Information.
  In the context of the mirror game, we use causation entropy to compute the net information flow among the players. Specifically, following the oCSE algorithm^32^ (see the Supplementary Information for details), given two players *i* and *j*, we compute $w_{ij}=C_{i\rightarrow j|(j,{\mathscr {P}}\setminus j)}$, where ${\mathscr {P}}$ is the set of players participating to the game.
  Then, for all players $i=1,\ldots ,N_{\mathrm{players}}$, we compute the outgoing and the incoming information flow as $\delta _{\mathrm{i, out}}= \sum _{j=1, j\ne i}^{N_{\mathrm{players}}}w_{ij}$ and $\delta _{\mathrm{i, in}}= \sum _{j=1, j\ne i}^{N_{\mathrm{players}}}w_{ji}$, respectively. Finally, we defined the *Net Causation Entropy* (${\text{NetCaus}}$) score for each player as
  8 $$\begin{aligned} {\text{NetCaus}}_i= \delta _{\mathrm{i, out}}- \delta _{\mathrm{i, in}} \quad \forall {\text{ i}} = 1,\dots ,\ N_{\mathrm{players}}. \end{aligned}$$
  ${\text{NetCaus}}_i$ quantifies the net information flow between player *i* and all the other players and therefore is taken as the *influence leadership index* in this study. Thus, the *influence leader* is defined as the participant with the highest value of ${\text{NetCaus}}$.

*Patterns identification* To unfold the emergence of the leadership patterns described in this paper, we combined the two indexes defined above. In particular, we noticed that the experimental trials could be collated into 4 different groups, one where no patterns emerged and the other three associated to each of the leadership scenarios described in the paper. In the first group, the participant who obtained the highest ${\text{NetCaus}}$ was also the person with the highest mean phase leadership index. In the second group the person with the highest ${\text{NetCaus}}$ was instead the one with the lowest value of the phase leadership index, while in the third two players, the ones with the highest and lowest phase pleadership index values, shared similar values of ${\text{NetCaus}}$.

To analyse each scenario, we removed outlier data from the data points. Then, for the first and second leadership scenario, we ran a first order (linear) polynomial regression, we checked the normality of residuals, the Pearson correlation coefficient and performed hypothesis tests on the regression coefficients (see Supplementary Information). For the third scenario, after verifying that a linear interpolation did not capture the data (the correlation was not significant), we then performed a second order polynomial interpolation.

*Level of coordination* As the joint task used as a paradigmatic case of study in the paper is oscillatory, we quantified the level of coordination among the agents by means of the *cluster phase* or *Kuramoto order parameter, z*, introduced for the first time by Kuramoto^57^. This index is defined as follows:

9 $$\begin{aligned} z_j(t_k)=\left| {\frac{1}{N_{\mathrm{players}}}} \sum _{i=1}^{N_{\mathrm{players}}} e^{j\theta _i(t_k)}\right| \quad \ \forall {\ j} = 1,\dots ,\ N_{\mathrm{trials}} \quad \ \forall {\ k} = 1,\dots ,\ N_{\mathrm{instants}}. \end{aligned}$$

This index is equal to 0 for pairwise agents in phase opposition and it becomes 1 when the players are perfectly overlapped in phase, reaching the highest level of coordination. We considered the mean value in the trial:

10 $$\begin{aligned} {{\bar{z}}}_j={\frac{1}{N_{\mathrm{instants}}}} \sum _{k=1}^{N_{\mathrm{instants}}} z_j(t_k) \quad \forall {\ j}\ =1,\dots ,\ N_{\mathrm{trials}}. \end{aligned}$$

An independent *t*-test was then calculated on these values to detect significant synchronization differences in the scenarios.

## Supplementary information

Supplementary Information 1.

## Data Availability

The authors declare that the data supporting the findings of this study are available in Repository with the DOI identifier 10.17605/OSF.IO/3DRFP (*Experiment1: Pendula*) and at the following link https://github.com/diBernardoGroup/LeadershipPatterns.git (*Experiment2: Chronos*).

## Code availability

The custom code for the analyses reported here is available from the corresponding author upon reasonable request.
