# Supplementary material for: Spontaneous emergence of leadership patterns drives synchronization in complex human networks
Source: Sci Rep. 2021 Sep 15;11:18379. doi: 10.1038/s41598-021-97656-y (PMC8443630; doi:10.1038/s41598-021-97656-y)
Supplement: Supplementary file 1 — Supplementary Information 1. [file 41598_2021_97656_MOESM1_ESM.pdf]

Supplementary Information

Spontaneous emergence of leadership patterns drives synchronization in complex human networks

Carmela Calabrese[1,2], Maria Lombardi[3], Erik Bollt[4], Pietro De Lellis[1,+], Benoît G. Bardy[2*,+], and Mario di Bernardo[1,5,*,+]

[1]Department of Electrical Engineering and Information Technology, University of Naples Federico II, Naples, 80125, Italy
[2]EuroMov Digital Health in Motion, University of Montpellier IMT Mines Ales, Montpellier, 34090, France
[3]Center for Robotics and Intelligent Systems (CRIS), Italian Institute of Technology (IIT), Genoa, 16163, Italy
[4]Electrical and Computer Engineering ECE Department, Clarkson University, Potsdam, NY 13699-5815, USA
[5]Department of Engineering Mathematics, University of Bristol, Bristol, BS8 1TR, UK
[*]mario.dibernardo@unina.it, benoit.bardy@umontpellier.fr
[+]these authors contributed equally to this work

Contents

A Dataset preprocessing and normality of the residuals **2**
B Additional analysis: distribution of leadership role across players **2**
C Further details on the oCSE algorithm used to compute the net information flow NetCaus **2**
D Supplementary Figures **4**
E Supplementary Tables **10**
References **11**

A Dataset preprocessing and normality of the residuals

In both Experiments 1 and 2 (see Figures S1 and S2 for further details on the two experimental setups), we split the trials in 4 different groups, that is, trials in which **Pattern 1**, **Pattern 2**, or **Pattern 3** was observed (groups from 1 to 3), and trials where no patterns were observed (group 4), see Tables S2 and S3. Then, we focused on groups 1 to 3 and, before running the linear regression analysis on the metrics, we removed the outliers from the distributions of the net information flow NetCaus and of the mean phase ranking $\overline{H}$. After performing the linear regression analysis, we tested for normality of the residuals through visual inspection of the QQ-plots and Lilliefors normality tests as detailed in Figures S3 and S4.

B Additional analysis: distribution of leadership role across players

The authors of[1] posited the existence of two diverse leadership structures. Specifically, in a group with a centralized leadership structure, a single member of the group acts as a leader, while all the other players enable and reinforce the leader identity. In opposition to this paradigm, leading actions can be distributed among group members across time. As we observed that the identity of the leaders emerging in the three patterns identified in the main text was not always the same, we used the Gini index[2] to discriminate between *distributed leadership* across the players or *centralized leadership*[1] emerged. For each of the six groups of participants (4 for Experiment 1, and 2 for Experiment 2, see *Methods* of the main manuscript), we focused on the trials where one of the three patterns was observed, and we computed for each player, say i, the percentage x_i of trials where s/he acted as a either a phase or an influence leader. The agents were indexed in non-decreasing order with respect to x_i, that is, for all i, $x_i \le x_{i+1}$. After normalizing such that $\sum_i x_i = 1$, we then obtained 6 distributions of x_i for each group, on which we computed the Gini index $0 \le I_g \le 1$, $g = 1, \dots, 6$, where the subscript g identifies the specific group considered. Originally introduced in economics to measure inequalities in the wealth distribution within a country, in this context the Gini index quantifies whether leadership is shared among the players, with $I_g = 1$ corresponding to a single agent consistently leading in all the trials, and $I_g = 0$ to all agents leading in the same number of trials. Specifically, for each of the six groups, the Gini index can be computed as

$$I_g = 1 - \frac{2}{N_{\text{players}} - 1}\left(N_{\text{players}} - \frac{\sum_{i=1}^{N_{\text{players}}} i x_i}{\sum_{i=1}^{N_{\text{players}}} x_i}\right). \tag{1}$$

In both experiments, we observe that the value of the Gini coefficient ranges between 0.50 and 0.63, thus suggesting that, although some players tends to lead more than others, leadership is generally shared among the group members.

C Further details on the oCSE algorithm used to compute the net information flow NetCaus

In the computation of transfer and causation entropy in the Methods of the main text, it is necessary to identify the correct delays in information processing, as highlighted in Ref. 3. In particular, they extended transfer entropy to account for the presence of multiple delays in source-target interactions. In fact, transfer entropy quantifies the information amount about the future of a stochastic process $Y(t_k)$ that the past of a second process $X(t_k)$ can provide in addition to the information already contained in the past of Y, see Eq. (6) in the main text. For this reason, we consider the following definition of causation entropy that accounts for heterogeneous information delays for the signal Y itself and the signals from the other

information sources:

$$C_{X\to Y|(Y,Z)} = H(Y(t_k)|Y(t_k-\delta),Z(t_k-\tau)) - H(Y(t_k)|X(t_k-\tau),Y(t_k-\delta),Z(t_k-\tau)), \quad (2)$$

where $Z(t_k)$ is a third process possibly encoding information on both X and Y, τ is the time required to process information on the other sources, and we delay the signal Y of δ seconds to predict its current state. In our work, the processes X, Y, and Z are the time-series of the positions of each player. Therefore, we set τ equal to 0.25 seconds, which is equal to 50 and 25 samples in Experiments 1 and 2, respectively, and corresponds to the average human response time in processing information[4–6]. As for the choice of δ, we observed the estimation of the causation entropy to be marginally affected by it. Indeed, we randomly picked a dataset (**CG1**, see the main manuscript) and estimated the causation entropy by varying δ between 0.05 and 0.25 seconds with step 0.05, and observed no significant difference in the emergence of the three leadership patterns highlighted in the main text, see Table S1. For this reason, we decided to select $\delta = \tau$ as proposed in Ref. 7, thus obtaining Equation (7) reported in the main text.

To compute causation entropy, the oCSE algorithm then uses the Kraskov-Strogbauer-Grassberger estimator[8] to estimate causation entropy from time-series. Specifically, it is a non parametric estimator based on κ-nearest neighbors. In this study, following[9], we selected κ as the square root of the sample size of the joint variables, which corresponds to setting $\kappa = 75$ in Experiment 1 and $\kappa = 55$ in Experiment 2. The computed causation entropy are then used to calculate the weights w_{ij}, see their definition after equation (7) of the main text. To determine whether or not these positive weights are significantly different from zero, shuffle tests with significance level $\alpha = 0.01$ in both the forward (discovery) and backward (removal) phases of the oCSE algorithm are used. We ran 100 trials in Experiment 1 and 500 trials in Experiment 2 per each hypothesis tests. The different number of trials is explained by the different length of the time series in the two experiments.

D Supplementary Figures

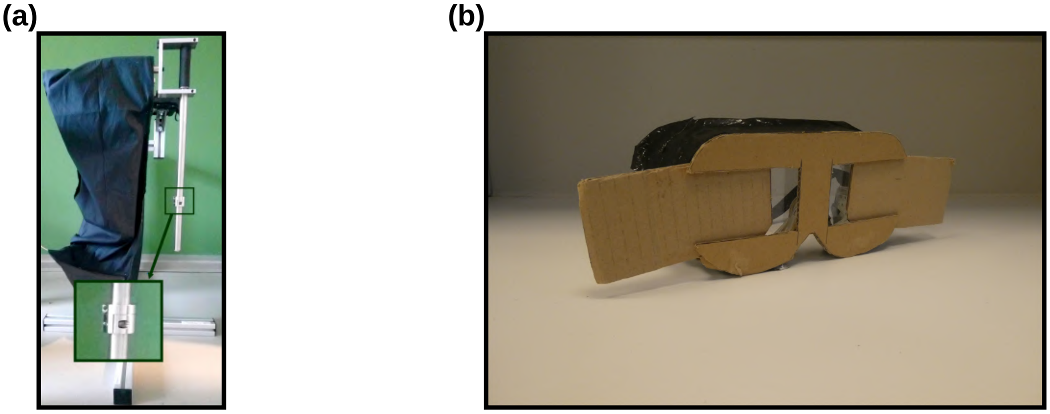

Figure S1. Experiment 1: Pendulum setup. Panel (a) shows the pendulum used to run experiments in the first experimental setting. Although masses could be added on the rod to modulate the inertia of the pendulum, we did not use this feature of the setup as we did not add any additional mass. Each participant was asked to wear handmade goggles (see panel (b)) to manipulate the field of vision and implement the different topologies of interest.

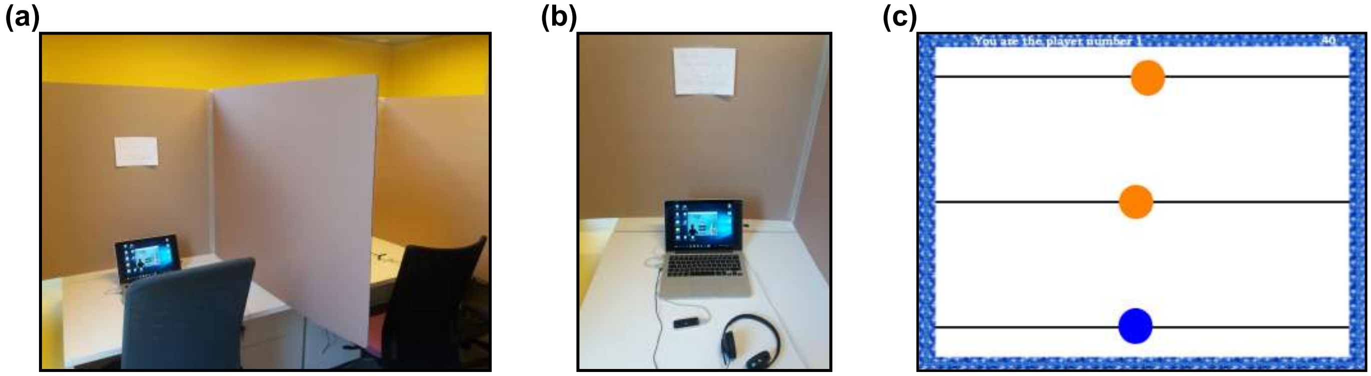

Figure S2. **Experiment 2: Chronos game setup.** Panel (a) shows the setup used to run experiments with the Chronos platform[10]. Cardboards were used to visually isolate participants and headphones playing white noise were during each trial to ensure acoustic isolation between participants. Each participant was sitting in front of a laptop pc equipped with headphones and a leap motion controller (as shown in panel (b)). Panel (c) shows the Chronos user interface where the blue circle represents user own motion, while the orange dots reflect the movement of the agents s/he is coupled with in the network structure.

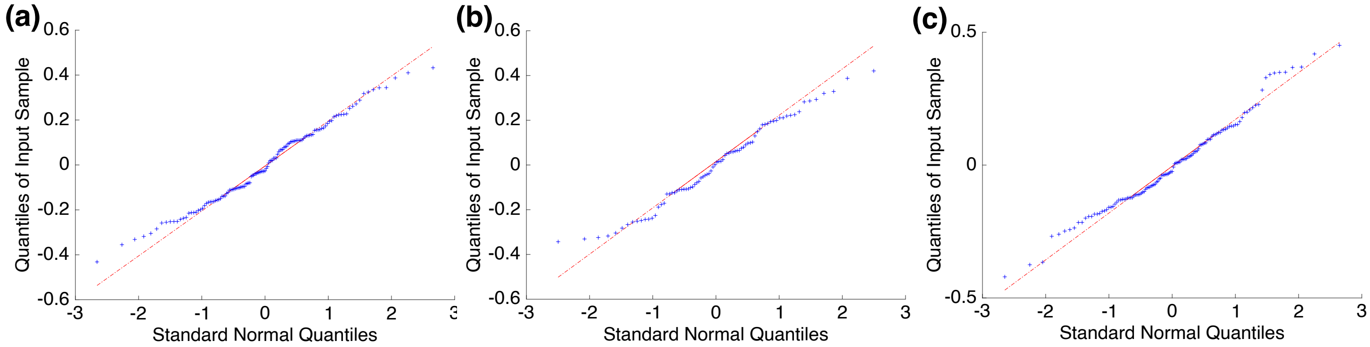

Figure S3. **QQplots of the residuals of a linear fitting of the pair NetCaus and $\overline{H}$ in Experiment 1.** Panels (a), (b), and (c) corresponds to trials where Patterns 1, 2, and 3 were observed, respectively. For each case, Lilliefors tests on residuals shows that the null hypothesis of normality of data cannot be rejected ($D(127) = 0.08, p = 0.06$ for Pattern 1, $D(80) = 0.07, p = 0.50$ for Pattern 2, and $D(123) = 0.06, p = 0.32$ for Pattern 3).

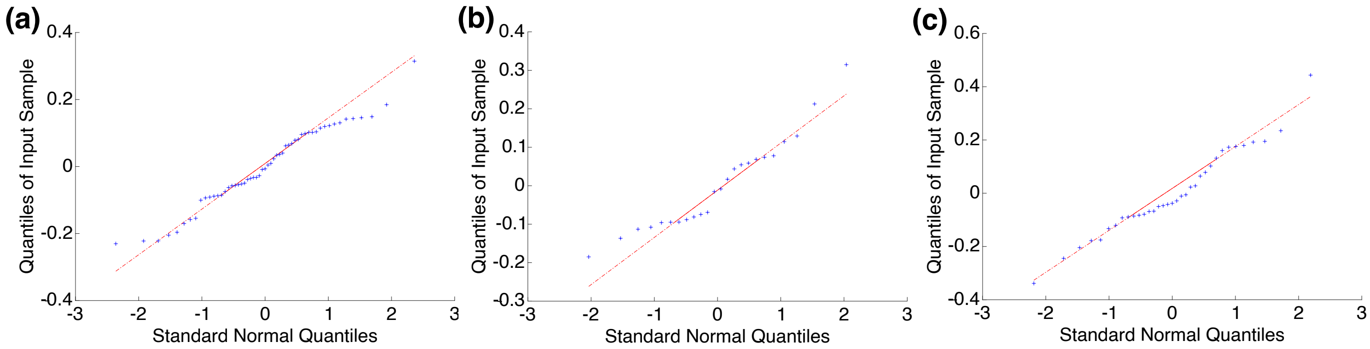

Figure S4. QQplots of the residuals of a linear fitting of the pair NetCaus and $\overline{H}$ in Experiment 2. Panels (a), (b), and (c) corresponds to trials where Patterns 1, 2, and 3 were observed, respectively. For each case, Lilliefors tests on residuals shows that the null hypothesis of normality of data cannot be rejected ($D(55) = 0.08, p = 0.49$ for Pattern 1, $D(24) = 0.18, p = 0.05$ for Pattern 2, and $D(35) = 0.11, p = 0.28$ for Pattern 3).

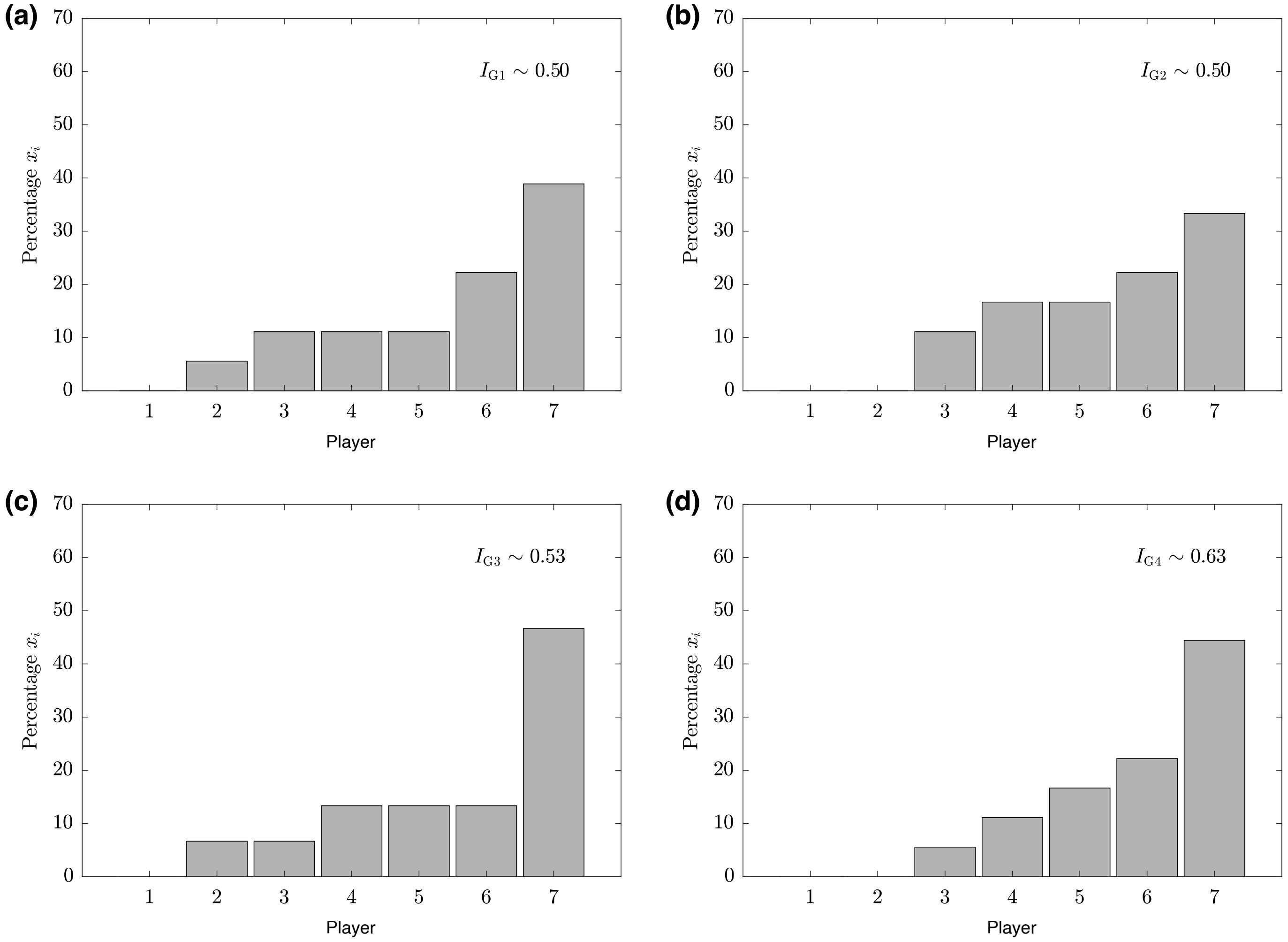

Figure S5. How leadership distributes among the players. Experiment 1: Pendula. Each panel shows the percentage x_i of trials where player i acted as a leader, for all $i = 1, \ldots, 7$. The agents are sorted so that x_i is non-decreasing. Panel (a), (b), (c), and (d) refer to group **G1**, **G2**, **G3**, and **G4**, respectively (see *Methods* in the main text for details on how players were clustered in each group). The value of the Gini index for each group is reported in the top-right corner of each panel.

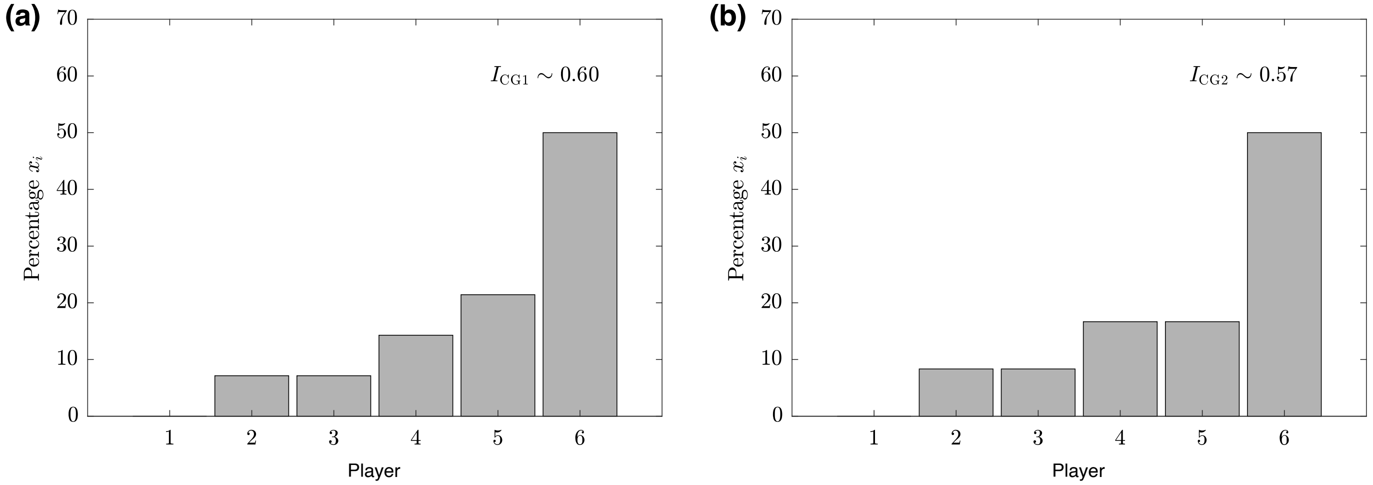

Figure S6. How leadership distributes among the players. Experiment 2: Chronos game. The two panels show the percentage x_i of trials where player i acted as a leader, for all $i = 1, \ldots, 6$. The agents are sorted so that x_i is non-decreasing. Panel (a) and (b) refer to group **CG1** and **CG2**, respectively (see *Participants* in *Methods* in the main text for details). The value of the Gini index values ($I_{\mathrm{CG1}} \sim 0.60$, $I_{\mathrm{CG2}} \sim 0.57$) given in the top-right corner of every panel suggests that there is no particular group member always assuming a leadership role but that in different trials different players took such role.

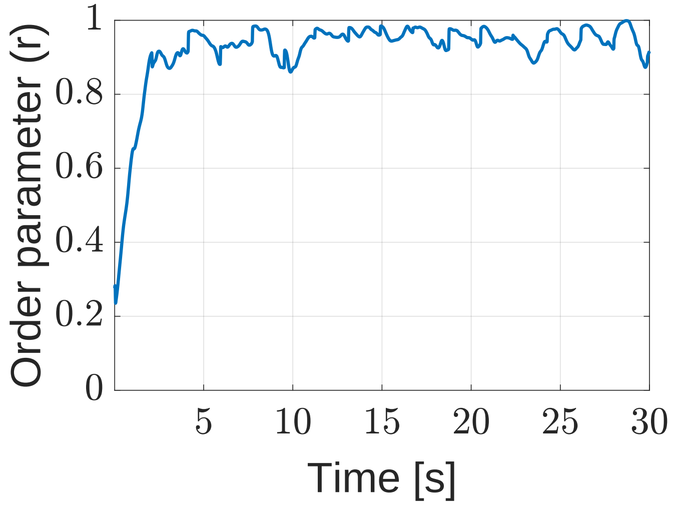

Figure S7. A representative example of time evolution of the phase synchronization level reached by the group along a trial. The panel shows the time evolution of group level of coordination r (see **Level of coordination** description in the *Methods* section in the manuscript). After about 5s, players achieve and maintain a good level of phase cohesiveness ($r \approx 0.95$ in average).

E Supplementary Tables

Self-prediction delay δ	Patterns	None	Pattern 1	Pattern 2	Pattern 3
$\delta = 0.05$	62.50%	37.50%	70%	10%	20%
$\delta = 0.10$	62.50%	37.50%	70%	10%	20%
$\delta = 0.15$	62.50%	37.50%	70%	10%	20%
$\delta = 0.20$	62.50%	37.50%	70%	10%	20%
$\delta = 0.25$	68.75%	31.25%	$\approx$ 55%	$\approx$ 18%	$\approx$ 27%

Table S1. Distibution of leadership patterns as function of self-prediction delay δ in the oCSE algorithm.

Topology	Pattern 1	Pattern 2	Pattern 3	None
Complete	25%	5%	50%	20%
Path	25%	15%	10%	50%
Ring	25%	5%	10%	60%
Star	20%	35%	25%	20%
Expected	11.90%	11.90%	4.77%	71.43%

Table S2. Distributions of the leadership Patterns in each topology. Experiment 1: Pendula. The last line represents the expected percentages if agents phase and influence leadership ranking are both extracted from a uniform distributions.

Topology	Pattern 1	Pattern 2	Pattern 3	None
Complete	37.5%	12.5%	25%	25%
Path	50%	0%	12.5%	37.5%
Ring	12.5%	0%	25%	62.5%
Star	25%	37.5%	12.5%	25%
Expected	13.33%	13.33%	6.67%	66.67%

Table S3. Distributions of the leadership Patterns in each topology. Experiment 2: Chronos game. The last line represents the expected percentages if agents phase and influence leadership ranking are both extracted from a uniform distributions.

References

1. DeRue, D. S. Adaptive leadership theory: Leading and following as a complex adaptive process. *Res. organizational behavior* **31**, 125–150 (2011).

2. Gini, C. reprinted in memorie di metodologia statistica, ed. *E. Pizetti & T. Salvemini (1955* (1912).

3. Wibral, M. *et al.* Measuring information-transfer delays. *PloS one* **8**, e55809 (2013).

4. Hanes, D. P. & Schall, J. D. Neural control of voluntary movement initiation. *Science* **274**, 427–430 (1996).

5. Schendan, H. E. & Ganis, G. Top-down modulation of visual processing and knowledge after 250 ms supports object constancy of category decisions. *Front. psychology* **6**, 1289 (2015).

6. Stanford, T. R., Shankar, S., Massoglia, D. P., Costello, M. G. & Salinas, E. Perceptual decision making in less than 30 milliseconds. *Nat. neuroscience* **13**, 379 (2010).

7. Lord, W. M., Sun, J., Ouellette, N. T. & Bollt, E. M. Inference of causal information flow in collective animal behavior. *IEEE Transactions on Mol. Biol. Multi-Scale Commun.* **2**, 107–116 (2016).

8. Kraskov, A., Stögbauer, H. & Grassberger, P. Estimating mutual information. *Phys. review E* **69**, 066138 (2004).

9. Duda, R. O., Hart, P. E. & Stork, D. G. *Pattern classification* (John Wiley & Sons, 2012).

10. Alderisio, F., Lombardi, M., Fiore, G. & di Bernardo, M. A novel computer-based set-up to study movement coordination in human ensembles. *Front. psychology* **8**, 967 (2017).
